# The administration route of indigestible gut permeability markers modulates urinary marker recovery in calves

**DOI:** 10.3168/jdsc.2024-0616

**Published:** 2024-09-02

**Authors:** C. Barozier, J.N. Wilms, J. Echeverry-Munera, D.J. Seymour, L.N. Leal

**Affiliations:** 1 Trouw Nutrition Research and Development, 3800 AG, Amersfoort, the Netherlands; 2 AgroParisTech, Université Paris-Saclay, 91120 Palaiseau, France

## Abstract

Indigestible gut permeability markers are used to assess gut integrity and can be administered to calves via a milk meal (MM) or orally pulsed (OP). This study investigated how marker administration route (ADM_R) affects the estimation of gut permeability in relation to milk replacer (MR) fat inclusion. Thirty-two newborn Holstein calves were blocked based on their arrival sequence at the facility. Within each block of 4 calves, calves were randomly assigned to one of 2 treatments (n = 16/treatment): a MR high in lactose (HL) and a MR high in fat (HF). During the first 5 d, calves were fed 6.0 L/d followed by 7.0 L/d offered in 2 meals per day at 15% solids. To evaluate gut permeability, indigestible markers (lactulose, d-mannitol, and chromium [Cr]-EDTA) were administered in 2 periods, on Tuesday and Thursday in the third week after arrival. Within each block, calves with different MR treatments were randomly assigned to a different marker ADM_R order: MM in the first period and OP in the second (n = 16) or the opposite order (n = 16). Thus, one block of 4 calves included all combinations of MR and ADM_R. Following marker administration, urine was collected over 2 sampling periods: the first from 0 to 6 h and the second from 6 to 24 h. Measurements included weekly BW and daily MR intake and fecal scoring. Intake, growth, and fecal consistency were not affected by dietary treatments or by ADM_R. The urinary recovery of lactulose was greater in calves fed HF during the 6- to 24-h and the overall 24-h collection period. Consistently, d-mannitol recovery tended to be greater in calves fed HF during the 24-h collection. With OP administration, the urinary recovery of all markers was greater between 0 and 6 h, whereas in the 6- to 24-h period, the recovery of lactose and d-mannitol were lower. Over 24 h of collection, Cr-EDTA recovery was greater with OP. No interaction between ADM_R and MR composition was detected. This suggests that both ADM_R were equivalent in assessing the effects of dietary interventions on gut permeability. However, different ADM_R likely affected the trajectory and time spent by the markers in each segment of the gut.

## Highlights

• Calves fed high-fat MR had increased recovery of gut permeability markers.
• Markers via milk meals or oral pulses yielded consistent measures of gut permeability.
• Different administration methods may affect marker trajectory through the gut.

## Graphical Abstract

**Figure:**
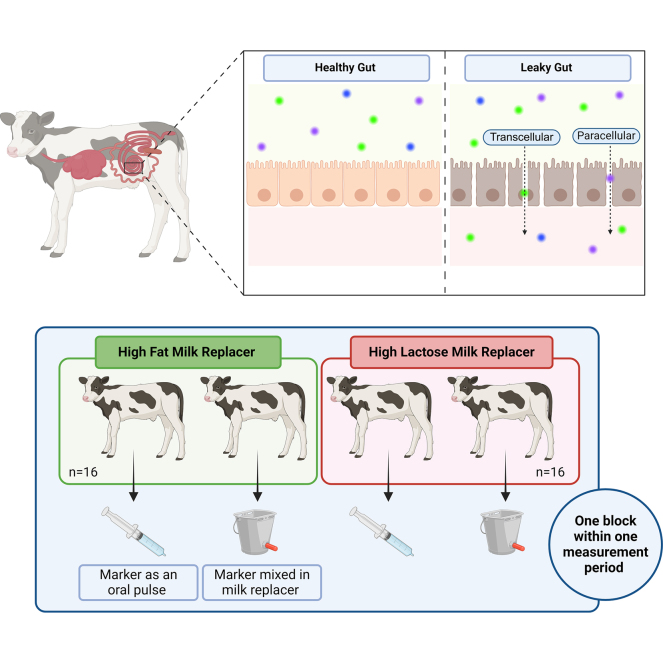
**Summary:** This study investigated how the marker administration route would affect the estimation of gut permeability in 32 Holstein calves fed a milk replacer (MR) high in fat (HF) or lactose. Indigestible permeability markers (lactulose, D-mannitol, and Cr-ethylenediaminotetraacetate) were administered either via the morning milk meal or orally pulsed. Calves fed HF exhibited higher lactulose and D-mannitol recovery over 24 hours of urine collection. Administration by oral pulse resulted in higher marker recovery over the first 6 hours of urine collection. No interaction was observed between the marker administration route and MR composition. Both administration techniques were consistent in assessing the effect of MR fat inclusion on gut permeability, albeit affecting the marker trajectory and transit time in the gastrointestinal tract.

The gastrointestinal tract (**GIT**) is a permeable and highly selective barrier allowing the efficient passage of nutrients while restricting the entry of unwanted compounds into circulation (Bischoff et al., 2014; Schoultz and Keita, 2020). Increased gut permeability, also known as leaky gut (Vanuytsel et al., 2021), has been linked to a variety of health disorders, such as Crohn's disease (Püspök et al., 1998) and type II diabetes in humans (Horton et al., 2014). Indigestible gut permeability markers enable noninvasive evaluation of gut integrity by entering the bloodstream upon absorption and being excreted in the urine, allowing marker recovery to be assessed as a percentage of oral dose (Schoultz and Keita, 2020; Vanuytsel et al., 2021). Large-sized molecules, including chromium (Cr)-EDTA (292 Da) and lactulose (342 Da), reflect paracellular permeability of the intestinal wall (Bischoff et al., 2014; Horton et al., 2014). The lactulose to d-mannitol ratio (182 Da) evaluates the intestinal permeability (**IP**) index, with impaired gut integrity indicated by increased urinary lactulose and decreased d-mannitol recovery (Vogelsang et al., 1998).

In cattle, gut barrier function is compromised during heat stress (Wang et al., 2020; Fontoura et al., 2022), feed deprivation or restriction (Zhang et al., 2013; Pisoni et al., 2022), ruminal (Khafipour et al., 2009; Minuti et al., 2014) and hindgut acidosis (Abeyta et al., 2023), diarrhea (Klein et al., 2008), and weaning (Wood et al., 2015). Furthermore, factors related to milk replacer (**MR**) composition modulate gut permeability in calves. Increased osmolality (Wilms et al., 2019) and fat inclusion in MR (Amado et al., 2019; Welboren et al., 2021) increased gut permeability to Cr-EDTA and lactulose. However, it is unclear what different degrees of permeability mean to calves in terms of health and performance, as there is no threshold for IP.

A commonly used method to administer ingestible permeability markers in calves is to mix the markers in a milk meal and subsequently collect urine over 12 to 24 h (Branco Pardal et al., 1995; Wilms et al., 2019; Welboren et al., 2021). However, in human medicine, the markers are administered separately from food after overnight fasting to avoid interaction with digestion processes and nutrient absorption (Vogelsang et al., 1998). Another method involves the administration of markers as an oral dose, instead of or before a milk meal (Klein et al., 2007, 2008). Studies on fat inclusion in MR show inconsistencies in results based on the method of marker administration. Amado et al. (2019) and Welboren et al. (2021) observed greater differences in marker recovery between high-fat (25%) and low-fat (18%) MR when markers were given via the MR, whereas Wilms et al. (2024a) reported smaller differences when the markers were administered as an oral pulse on empty gut (25% vs. 18% fat). This led to the question of whether MR high in fat increases gut permeability or if the interactions between gut permeability markers and the absorption of nutrients in the meal created a biased evaluation. Therefore, it was hypothesized that feeding an MR high in fat would increase marker recovery compared with an MR high in lactose when markers are mixed with the morning milk meal, but not when markers are orally pulse-dosed. Thus, this study aimed to investigate the routes of administration of indigestible permeability markers, as well as to determine whether fat inclusion in MR modulate gut permeability in calves.

This study was conducted at the Calf Research Facility of Trouw Nutrition R&D (Boxmeer, the Netherlands) between September and October 2021. All procedures described in this article complied with the Dutch Law on Experimental Animals, which complies with ETS123 (Council of Europe 1985 and the 86/609/EEC Directive; Council Directive 86/609/EEC, 1986) and were approved by the animal welfare authority (Centrale Commissie Dierproeven, CCD, Utrecht, the Netherlands).

A power analysis was conducted with a power of 80%, and an α level of 0.05. Based on Amado et al. (2019), with 4-wk-old calves fed a restricted allowance of an MR high in fat (n = 18) or high in lactose (n = 14), a standard deviation of 0.586% was assumed for Cr-EDTA recovery over 24 h of urine collection with a minimal meaningful difference of 0.58%. Therefore, the minimal sample size to detect differences under this assumption was calculated to be 16 calves per treatment.

A total of 32 apparently healthy male Holstein calves were collected from collaborating dairy farms 24 to 72 h after birth. A standardized colostrum protocol was followed on the farm of origin, which included 3 feedings of colostrum in the first 24 h: 3.0 to 4.0 L within 1 h after birth, followed by 2 feedings of 2.0 L. Colostrum quality (>22% Brix) was measured using a Brix refractometer (model RHB-32ATC; Westover Scientific, Mill Creek, WA). Calves were then fed 2 meals of 3.0 L of bulk tank whole milk (**WM**) twice daily at the farm of origin following the regular feeding schedule. Both colostrum and WM were fed at 40°C using a bottle or teat bucket. Successful application of the colostrum protocol was evaluated upon arrival to the research farm (24–72 h after birth), by measuring serum IgG using a portable Multi-Test Analyzer (DVM Rapid Test II, Vetlab, Palmetto Bay, FL).

Calves were blocked based on the sequence of arrival at the research facility. Within each block of 4 calves, calves were randomly assigned to a dietary treatment (n = 16 calves per treatment) including a MR high in fat (**HF**; Sprayfo Delta, Trouw Nutrition, the Netherlands) with 25% fat, 40% lactose, and 22.5% CP (% DM basis) or a MR high in lactose (**HL**; Sprayfo Royal, Trouw Nutrition, the Netherlands) with 18% fat, 44% lactose, and 22.5% CP. The randomization was performed using [RANDBETWEEN (0,100000)] in Microsoft Excel (Microsoft Corp.). The HF MR (397 mOsm/kg) was composed of 50.0% skim milk powder, 25.0% vegetable fats, 16.0% whey powder, 6.0% whey protein concentrate, 2.0% wheat starch, and 1.0% premix (vitamins, minerals, and additives). The HL MR (456 mOsm/kg) was composed of 50.0% skim milk powder, 18.0% vegetable oils, 23.0% whey powder, 6.0% delactosed whey powder, 2.0% dextrose, and 1.0% premix. In both treatments, the fat blend consisted of 65% palm and 35% coconut fat. The MR was reconstituted at 150 g/L (15.0% solids). Therefore, the MR diets were not isoenergetic because fat is more energy-dense than carbohydrates. This led to a ME content of 20.6 MJ/kg DM for HF and 19.1 MJ/kg DM for HL.

Lactulose (0.2 g/kg BW; Sigma-Aldrich, Zwijndrecht, the Netherlands) and d-mannitol (0.12 g/kg BW; Sigma-Aldrich) were dissolved separately in 100 mL of warm water. In addition, a liquid Cr-EDTA (9.5 g/L Cr-EDTA solution; MasterLab, Boxmeer, the Netherlands) was adjusted for each calf to provide a dose of 0.1 g/kg BW of Cr-EDTA (Wilms et al., 2024a). Gut permeability markers were administered over 2 periods during the third week after arrival. Each pair of calves with different MR treatments within a block of 4 calves was randomly assigned to a different gut permeability marker administration route (**ADM_R**) order using the [RANDBETWEEN (0,100000)] in Microsoft Excel. Thus, one block of 4 calves included all combinations of MR and marker ADM_R. On the first measurement day (Tuesday, 13.8 ± 1.2 d after arrival), 2 calves within each block of 4 received the markers mixed with the morning milk meal (**MM**) at 0630 h, whereas the other 2 calves received the markers orally pulse (**OP**) dosed. Calves receiving the markers as an OP were feed-deprived for 6-h period and received their morning MR meal 6 h later, at 1230 h. On the second measurement day (Thursday, 17.1 ± 1.3 d after arrival), calves that received the markers mixed with the morning MM, received the markers as an OP dose and vice versa. The time elapsed between the 2 periods was determined based on Zhang et al. (2013), where 48 h was necessary to eliminate 98% of Cr-EDTA.

Calves were housed indoors in individual pens (2.67 m^2^) separated by galvanized bar fences and equipped with rubber-slatted floor in the front area and a lying area in the back, which contained a mattress covered with flax straw. Total urine collection was performed as described by Wilms et al. (2019). Calves were fed 6.0 L in 2 equal-sized meals at 0630 and 1700 h through clean teat buckets from d 1 to 5 after arrival and 7.0 L thereafter. Calves had ad libitum access to water and chopped straw available in plain buckets and refreshed daily.

Calves were weighed upon arrival and then once weekly at 1100 h with a custom scale (W2000; Welvaarts Weegsystemen, Hertogenbosch, the Netherlands). Individual feed and water intakes were manually recorded daily by weighing the unconsumed amounts. General health was monitored daily by caretakers, and the administration of medical treatments was recorded daily. Fecal scoring was performed daily by taking photos of feces after the morning meal. Pictures were scored as 0 (no diarrhea, normal to pasty consistency) or 1 (diarrhea, loose/watery feces) by an examiner who was blinded to the dietary treatments. On the day before each urine collection period, baseline urine samples were collected using plastic bags attached to the calf. Following marker administration, urine was quantitatively collected over 2 collection periods, in which 2 samples were subsequently taken from the urine collection after thorough mixing: from 0 to 6 h and from 6 to 24 h relative to marker administration. Urine collection bags were attached to the calves using medical glue, as described by Wilms et al. (2020).

Lactulose, d-mannitol, and Cr in urine were measured by MasterLab (Boxmeer, the Netherlands) as described by Mellors et al. (2023) and Wilms et al. (2024a). The concentrations of Cr-EDTA, d-mannitol, and lactulose in the urine were multiplied by the volume of urine of the corresponding collection period to estimate the excreted mass of each marker. To obtain the percentage of marker recovery in urine, the marker weight was divided by the weight of the marker ingested for d-mannitol and lactulose and by the volume of Cr-EDTA solution multiplied by the concentration of Cr in the Cr-EDTA solution.

Analyses of variance for continuous outcomes were performed using the MIXED procedure of SAS v9.4 (SAS Institute Inc., Cary, NC). The effects of MR treatment, ADM_R, and the interaction between them were considered fixed, whereas the effect of the block was considered random. The effects of both the animal and period were not statistically different than zero and were subsequently removed from the model. When analyzing BW, the effects of time and all treatment-by-time interactions were included as fixed effects, with initial BW included as a covariate, and repeated measures modeled using a first-order autoregressive covariance structure. The Kenward-Roger correction was used to compute the adjusted denominator degrees of freedom for tests of fixed effects. The effect of MR treatment on fecal scores was analyzed with a β-distribution and logit link function using the GENMOD procedure in SAS (SAS Institute Inc., 2018). Statistical significance was declared at *P* ≤ 0.05, and trends toward statistical significance were reported when 0.05 < *P* < 0.10.

No differences between calves fed HF and HL were observed on the arrival parameters including the age at arrival (3.1 ± 0.20 d) and serum IgG (1,861 ± 130.2 mg/dL). Milk replacer intakes did not differ between HF and HL, and by design, ME intakes were higher in calves fed HF than HL. Body weight at arrival (45.8 ± 0.96 kg) and final BW at wk 3 (53.1 ± 0.38 kg) were not different between calves fed HF and HL (*P* = 0.57). Similarly, the percentages of diarrheic calves at wk 1 (41.6%), wk 2 (46.3%), and wk 3 (24.4%) were comparable.

Concentrations of all indigestible markers in baseline urine samples were below 0.01 g/L for each sampling period, which is considered negligible. The marker recovery of lactulose was greater in calves fed HF during the 6- to 24-h collection period (*P* = 0.03; Table 1) and in the overall 24-h collection period (*P* = 0.01). Consistently, d-mannitol recovery tended to be greater in calves fed HF during the 24-h collection period (*P* = 0.07). The IP index was higher in calves fed HF during the 0- to 6-h period (*P* = 0.02) and higher in calves fed HL during the 6- to 24-h period (*P* = 0.04). However, when considering the whole 24-h collection, no differences in IP index were detected. In contrast, the recovery of Cr-EDTA was not affected by the dietary treatments. With OP administration, the recovery of lactulose (*P* < 0.01), d-mannitol (*P* < 0.01), and Cr-EDTA (*P* < 0.01) was greater between 0 and 6 h, whereas in the 6- to 24-h period, the recovery of lactose (*P* = 0.04) and d-mannitol (*P* = 0.01) was lower. The IP index was greater when the markers were orally pulsed, but this was only the case in the first 6 h of urine collection (*P* < 0.01) and not thereafter. Over 24-h collection, Cr-EDTA recovery was greater with OP (*P* = 0.02). No interaction between ADM_R and MR composition was detected.

**Table 1:** Gut permeability assessed by fractional urinary recovery of indigestible permeability markers in 3-wk-old calves

Marker recovery^2^	Treatment^1^				Pooled SEM	*P*-value		
	MM	OP	HF	HL		ADM_R	MR	MR × ADM_R
Lactulose								
0–6 h	0.72	1.62	1.32	1.01	1.170	<0.01	0.15	0.97
6–24 h	1.66	1.22	1.68	1.20	1.439	0.04	0.03	0.95
0–24 h	2.37	2.84	3.00	2.21	2.607	0.13	0.01	0.99
d-Mannitol								
0–6 h	2.50	4.02	3.50	3.03	3.263	<0.01	0.31	0.36
6–24 h	5.12	3.44	4.77	3.79	4.28	0.01	0.13	0.54
0–24 h	7.58	7.47	8.27	6.78	7.522	0.29	0.07	0.89
IP index^3^								
0–6 h	0.296	0.388	0.376	0.308	0.0207	<0.01	0.02	0.32
6–24 h	0.590	0.652	0.458	0.784	0.1504	0.69	0.04	0.22
0–24 h	0.385	0.484	0.373	0.496	0.0902	0.33	0.22	0.89
Cr-EDTA								
0–6 h	1.51	3.25	2.43	2.33	2.378	<0.01	0.80	0.31
6–24 h	4.00	3.71	4.05	3.66	3.855	0.52	0.38	0.52
0–24 h	5.51	6.96	6.48	5.98	6.232	0.02	0.42	0.26

1 Marker recovery was measured in calves fed an MR high in fat (HF, n = 16) or an MR high in lactose (HL, n = 16). The administration route (ADM_R) of the marker was either via the morning milk meal (MM; n = 34) or orally pulsed (OP; n = 34). Markers were administered twice on Tuesdays and Thursdays in wk 3 after arrival, thus ensuring a washout period of 24 h. The ADM_R on Tuesday was MM for half of the calves and OP for the other half; the opposite was true on Thursday.

2 Expressed in % oral dose.

3 The intestinal permeability (IP) = ratio between urinary recovery (%) of lactulose and d-mannitol.

Increased gut permeability marker recovery in relation to fat inclusion in MR has been previously observed when markers were mixed into the morning meal (Amado et al., 2019; Welboren et al., 2021) and orally pulsed instead of the morning meal (Wilms et al., 2024a). The higher IP index in calves fed with HF within the first 6 h was consistent with this observation. However, the higher IP index in calves fed HL in the 6- to 24-h collection period may indicate different marker excretion rates within different collection periods. When administered in the milk meal, Amado et al. (2019) speculated that the absorption of indigestible markers could interact with absorption of dietary fats leading to enhanced maker absorption. This was based on studies performed in rodents and humans, in which high dietary fat has been associated with decreased intestinal integrity (Boutagy et al., 2016). Indeed, intestine-derived LPS can be incorporated into micelles and chylomicrons and absorbed alongside dietary lipids, transiently increasing its appearance in the blood (Boutagy et al., 2016). In contrast, when markers are orally pulse-dosed instead of the morning meal, leaving a 6-h period in which calves are feed-deprived allows solely focusing on maker absorption on an empty gut. The lack of interaction between MR composition and marker ADM_R therefore suggests that high fat inclusion in MR increases gut permeability. Previous studies have shown that dietary fat can decrease intestinal integrity in rats, and which could be related to changes in mRNA expression of tight junction genes (Suzuki and Hara, 2010; Boutagy et al., 2016). However, these changes have only been associated with very high fat diets; in the present study, fat inclusion remained lower than in bovine WM. Increasing the inclusion of fats differing from bovine milk fat in fatty acid profile and triglyceride structure might also lead to increased gut permeability. However, recent research (Wilms et al., 2024b; Wilms, 2024) has shown that fat composition in MR did not affect gut permeability, even when compared with bovine milk fat. While it is possible that low-quality fats could impair gut permeability, this has not yet been studied in calves.

When the indigestible permeability marker solutions are orally pulsed instead of the morning meal, it is likely that these marker solutions do not induce the closure of the esophageal groove and thus may enter the reticulorumen. Therefore, oral pulse dosing markers may not solely represent IP. In contrast, when markers are mixed with a milk meal, the closure of the esophageal groove delivers the milk directly into the abomasum. According to Penner et al. (2014), the jejunum of Holstein steer calves has the highest transcellular permeability, whereas the omasum has the greatest permeability to larger-sized molecules, followed by the rumen and the jejunum. Therefore, in 3-wk-old calves, which can be considered functionally monogastric, it can be hypothesized that the jejunum is the main site of paracellular permeability. However, it is possible that the greater urinary marker recovery observed in the first 6-h collection postadministration when the markers were orally pulsed could be due to a larger absorptive surface in the rumen and intestines. This would also explain why Cr-EDTA recovery was greater in the 24-h urine collection in calves receiving the markers as an oral dose rather than mixed with the MR. Nevertheless, the similar marker recovery over a 24-h collection period for lactulose and d-mannitol did not support this hypothesis. It is important to note that based on the ADM_R, indigestible permeability markers do not remain in each segment of the GIT for the same time. When administered in the morning milk meal, the markers are likely to remain longer in the abomasum and then in the intestines compared with oral pulse administration. This is because abomasal emptying in calves can take between 129 and 206 min to be completed (Burgstaller et al., 2017), and thus, for a milk meal to be released from the abomasum to the intestines. This could explain why for the 0- to 6-h collection period, the urinary marker recovery was higher for all markers when orally pulsed than when administered via the meal. In addition, the higher IP index observed in calves receiving the marker as an oral pulse dose within the first 6 h of urine collection may stem from a faster excretion rate than when the markers are mixed with milk. In contrast, in the 6- to 24-h collection period, the urinary recovery of lactulose (only with HL MR) and d-mannitol was higher when the markers were mixed with milk than when administered as an oral pulse dose. In view of these results, it can be suggested that the urine collection period should be adapted to the administration technique used, limiting the collection to 12 h when the markers are orally pulsed. However, further investigations are needed to determine the exact trajectory and the time spent by indigestible permeability markers in each segment of the GIT, depending on the ADM_R. It should also be noted that when markers were administered OP, the meal was given 6 h after the usual time, and feed deprivation is known to increase gut permeability due to stress (Zhang et al., 2013; Pisoni et al., 2022).

The initial hypothesis that feeding an MR high in fat would increase marker recovery as compared with an MR high in lactose when markers are mixed with the morning milk meal, but not when markers are orally pulse-dosed, was not verified. These results suggest that higher fat levels in MR increase gut permeability when nutrient absorption is taking place, but also on an empty gut. In addition, the ADM_R of indigestible gut permeability markers modulated urinary marker recovery during different collection periods. When administered into the milk meal, the marker recovery was higher in the 6- to 24-h period, whereas when administered as an oral dose, the marker recovery was higher in the 0- to 6-h period.
